# Digital Phenotyping Reveals Phenotype Diversity and Epistasis among White Spotting Alleles in the American Paint Horse

**DOI:** 10.3390/genes14112011

**Published:** 2023-10-27

**Authors:** Chelby Lynn Gossett, Danielle Guyer, Jessica Hein, Samantha A. Brooks

**Affiliations:** 1 UF Genetics Institute, University of Florida Department of Animal Sciences, Gainesville, FL 32611-0910, USA; 2 American Paint Horse Association, Fort Worth, TX 76161-0023, USA

**Keywords:** KIT, MC1R, coat color, epistasis, breed registry

## Abstract

White spotting is an iconic feature of the American Paint Horse. The American Paint Horse Association (APHA) is dedicated to recording pedigree and performance of this stock-type breed, while preserving its distinctive coat color and conformation. Here, the depigmented proportion of the coat (% white coat) was measured using digital photograph analysis of 1195 registered American Paint Horses. Genotypes for nine white-spotting polymorphisms commonly found in Paint Horses, and two pigment-producing loci *MCIR* and *ASIP* genes, were also provided by the APHA. White-coat percent significantly increased in horses with more white-spotting alleles present, regardless of the number of loci bearing those alleles, likely due to a strong additive genetic effect at each white-spotting locus, as well as an additive epistatic effect among white spotting loci. Paint Horses with a chestnut base coat color (genotype *e/e* at *MC1R*) possessed a significantly higher white coat percentage, suggesting confirming an epistatic interaction between pigmentation signaling genes and loci for white spotting. The APHA registry categories of Regular versus Solid Paint-Bred also differed in their median white coat percentage (*p* < 0.0001), but not in the overall ranges of this phenotype, reenforcing the importance of the regional patterns of the depigmentation in the definition of the desired APHA phenotype. Multi-locus phenotype prediction models for white-coat percentage performed only moderately well, and improvements in the sample size and the number of loci genotyped will likely be needed before such an approach could be used practically by APHA breeders. In the future, models that enable phenotype prediction based on genotypes, and automated phenotype assessment could increase the production of valuable visual traits in the American Paint Horse population and improve the APHA member experience during the registration process.

## 1. Introduction

The American Paint Horse (APH) is a stock-type breed of horse well-known for its colorful coat and versatility. Today, the American Paint Horse can be seen competing in a variety of all-around events such as reining, western pleasure riding, hunt seat and working cow horse [1]. The breed is registered through the American Paint Horse Association (APHA), founded in 1965 as a consolidation of the American Paint Stock Horse Association and the American Paint Quarter Horse Association to preserve the breed’s coat color, conformation, and utility. As the world’s second largest equine-breed association, APHA currently has over 1.1 million horses registered [2]. In the American Paint Horse market, white-spotting phenotypes are desirable and on average doubles the value of an individual foal [3]. Thus, preserving white-spotted coat patterns in the APHA is an economic driving force for the association membership.

The APHA uses photographs to visually assess spotting phenotype and based on standardized thresholds determines the placement of each horse into one of two categories: the Regular Registry or the Solid Paint-Bred Registry [1]. APH registered with the Regular Registry must have their parentage verified and meet the color or generational requirements. Color requirements for the Regular Registry state that each horse must possess a “natural Paint marking”, or solid white hair with underlying unpigmented skin, present from birth, and extended at least two inches beyond one of three reference points defined by the APHA [1]. These thresholds include a reference point on the head and two on the at the knee or hock of the horse. In cases where the degree of white spotting on the horse coat does not exceed the APHA-determined thresholds for entry into the Regular registry, phenotype and genotype are also inspected and can, as of 2017, gain Regular Registry status for a horse that possess an approved genotype for white spotting alleles [1]. Horses that meet select parentage requirements but don’t otherwise meet requirements for Regular Registry based on the degree of white spotting on the coat, or have an applicable white spotting genotype(s), can advance to the Regular Registry upon certain conditions, but their pattern type remains designated as “solid” rather than “overo,” “tobiano,” or “tovero” [1]. This option did not affect horses in the study, as the rule change occurred after data collection.

Here we examine nine white spotting loci genotyped using a commercial service during the APHA registration process. Tobiano (*TO*) results from an inversion of approximately 100 kb near the *KIT* gene, likely results in a white spotting pattern by disrupting regulatory sequences important for controlling transcription of *KIT* [3]. Sabino (*SB1*) is a white spotting pattern produced by a single nucleotide polymorphism (SNP) within intron 16 leading to the skipping of exon 17 in the *KIT* gene [4]. Overo/Overo Lethal White Syndrome (*O*) results in a white spotting pattern in the heterozygous state, but homozygous individuals are completely white and die within days of birth and is associated with a mutation in the endothelin-B receptor gene (*EDNRB*) [5]. We observed just three of the alternative alleles for the “variable white” group of spotting patterns at the *KIT* locus. The *W5* allele is associated with a 1-bp deletion in exon 15 while the *W10* allele is attributed to a 4-bp deletion within exon 7 [6]. The *W20* allele is associated with a missense variant in exon 14 of *KIT* [7]. We previously documented that the *W20* allele within the *KIT* gene is strongly associated with the white-spotting phenotype as defined by the APHA [2]. There are three alleles for the “splashed white” group of white spotting patterns, two within the *MITF* gene (SW1 and SW3) and one within the *PAX3* gene (SW2). *SW2* is associated with a missense mutation on *PAX3*, *SW1* with a 10 bp insertion on a *MITF* promoter, and *SW3* with a frameshift mutation in *MITF* [8]. Homozygous *W5*, *W10*, and *SW3* genotypes are presumed to be lethal [6,8]. *MITF- SW5*, *KIT- W22* and many other recently discovered white spotting alleles were not examined here as these tests were not yet commercially available when the study began. A comprehensive and well-curated table of alleles in the horse at each of these loci is maintained at the Online Mendelian Inheritance in Animals database (OMIA.org, accessed on 15 October 2023 [9]).

Objective, quantitative methods of phenotyping white spotting patterns in domesticated species are not currently employed and commonly this is accomplished visually using non-empirical, qualitative thresholds based on breed standards [10,11]. Additionally, although there are several of cases of atypical expression patterns noted in the literature [12,13], there are no reports estimating the frequency of various degrees of depigmentation for each of the known white spotting alleles. In this work we aimed to quantitatively evaluate the typical expression of these nine common white spotting alleles in the APH, investigate genetic interactions between pigmentation loci using these continuous phenotypes, and test predictive models for these phenotypes based on these genotypes. 

## 2. Materials and Methods

### 2.1. Records

The APHA provided data for 1195 registered Paint Horses including: registration number, registry information of the horse’s sire and dam, sex and age of the horse, and the registration date and type (‘Regular’ or ‘Solid Paint-Bred’). The APHA also supplied scanned photographs of each horse taken by the owner at the time of registration and used to define the registration type. 

### 2.2. Genotypes

The 1195 horses sampled from the years 2016 to 2020 were genotyped for nine coat color alleles for white spotting patterns by the Veterinary Genetics Laboratory, University of California, Davis as a commercial service provided to the APHA. The following alleles were examined at four specific loci: *KIT*-*TO*, *SB1*, *W5*, *W10*, *W20* or “*N*”, *EDNRB-O* or “*N*”, *MITF-SW1*, *SW3* or “*N*”, and *PAX3-SW2* or “*N*”. “*N*” refers to the wild-type (*wt*) allele as designated by the testing service provider. While there are other equine white-spotting pattern genetic variants recorded in the scientific literature, these nine are relevant and commonly used for the APHA. More details on these nine polymorphisms are recorded in our previous publication [2]. Alleles at the *MC1R* and *ASIP* loci were also genotyped as these loci may impact the expression of depigmentation phenotypes [2,9].

### 2.3. Digital Photo Analysis

Digital photos of 760 out of the 1195 submitted horses were of sufficient quality for analysis with the graphics editing software GNU Image Manipulation Program (GIMP) version 2.10.14 to determine the percent of white pixels within the area of the photo showing the coat of each horse. Many images had to be excluded from the study due to poor exposure and only partial lateral views of the horse. Within GIMP the Free Select Tool was used to hand-trace the outline of the horse’s body on a single lateral view of each horse. The horse’s tail, mane, forelock, and hooves, each a long-keratinized growth from a single location on the surface of the horse, were excluded so as not to overrepresent these surfaces in the final calculation of pigmented area. The full-body images were converted to a strictly black and white (1-bit) palette distinctly isolating, white-spotted regions from the visible surface bearing the underlying base coat pigmentation. White pixel count and total pixel count of the visible horse surface were recorded and used to calculate the total percentage of white coat. All photo processing and calculations were conducted by a single researcher, and then replicated by a second independent observer, to check each calculation for accuracy (interobserver variation in all photos was >5%). 

### 2.4. Statistical Analysis

All statistical tests were conducted in JMP version 16.1.0 (SAS Institute Inc. Cary, NC, USA) First, we examined the distribution of the percent white spotting for each of the categories, assessing goodness-of-fit to a normal distribution with the Shapiro wilk test. As all distributions were strongly skewed, we presented the median values for percent white spotting, and tested difference from the group comprising horses with none of the tested spotting alleles for each genotypic category using a Welch’s test. We did not conduct any comparisons to genotypic categories possessing less than 3 horses. In analysis examining the effect of *MC1R* and *ASIP* loci on the percent white spotting we excluded all horses carrying at least one *TO* allele, due to strong linkage between *KIT* and *MC1R* induced by the paracentric inversion defining the *TO* allele [2,3]. To test the relationship between the total number of spotting alleles and the percent white coat we used ANOVA and linear regression.

### 2.5. Predictive Modeling

We assessed the performance for four different models predicting the proportion of white coat phenotype using ten of the polymorphisms genotyped here (eight white spotting alleles, excluding *W10* as no horses were observed with this allele in the sample, and the *MC1R* and *ASIP* loci). We trained each model using genotypes and phenotypes from two-thirds of the data set (507 out of 760 horses) then measured validation statistics after applying the resulting model to the remaining 253 horses. First, to employ a Generalized Linear Model (GLM) we formatted all genotypes quantitatively using a 0,1,2 scheme, setting the wildtype as the “0” score. Then we assessed the effects of all individual loci, as well as the interaction of two-allele combinations, retaining the two-allele effect in the model if the interaction exceeded a *p* value of 0.01 (TO*O, TO*W20, TO*E, O*SB, O*W20, SB*A, and SW1*SW2). The final model included all ten individual loci, as well as the eight significant two-locus interactions. The linear regression (conducted with the genotypes coded as homozygous reference = 0, heterozygous = 1 and homozygous alternate = 2) of the actual phenotype by the predicted phenotype provided the correlation (R-squared) and the root mean square error (RMSE) rather than the root average square error (RASE) assessed for the other three models. For the Standard Least Squares (SLS) model included the same ten genotypes and eight two-genotype interactions but employed a categorical model for each of the genotypes (homozygous reference, heterozygous and homozygous alternate). 

For the remaining two approaches we also modeled the genotypes as categorical values at each locus and trained the model on the same set of 653 horses used for the GLM and testing each model in the remaining 322 horses but considered only the effect of the 10 individual white spotting and base coat color alleles. To apply a Bootstrap Forest, we considered models with 100 trees, a minimum split size of 5, minimum split number of 10 per tree, and sampling 8 terms per split. For the neural net model, optimization was performed by testing all possible models with one or two layers, 2–7 nodes per layer, and considered fits using the TanH, Linear and Gaussian structures. The final model possessing the lowest Root Average Square Error (RASE) value utilized two layers, with seven nodes in the first layer and three nodes in the second layer, both applying a TanH structure. 

## 3. Results 

All the heterozygous, single allele sample populations differed significantly in phenotype from the group of horses with no known alleles, including the *W20*/*N* genotype, a phenotype hotly debated among APHA members (Table 1). Among the single locus genotypes, the homozygous tobiano genotype (*TO*/*TO*) has the highest median proportion of white coat, closely followed by the heterozygous tobiano genotype (*TO*/*N*). The *TO*/*N* *W20*/*N* combination largest median white coat percentage of the groups with compound heterozygous genotypes. 

**Figure 1:**
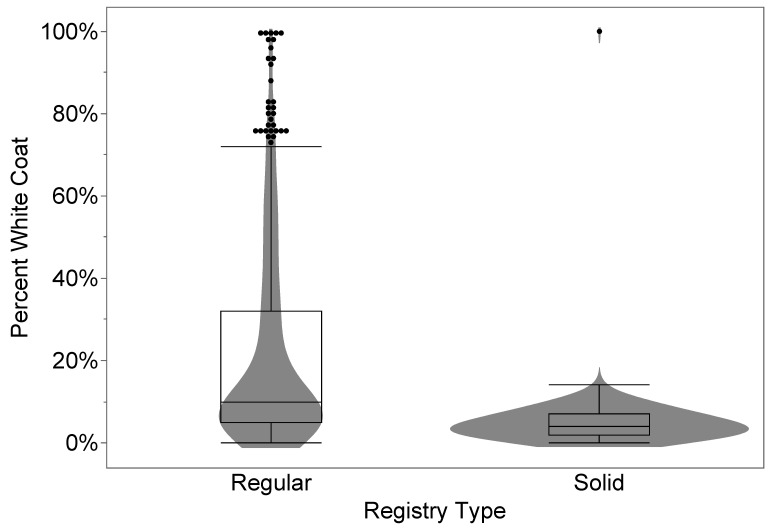
Percent white coat phenotypes among the two APHA-determined registration types reveal overlapping ranges, though the medians differ significantly (*p* < 0.0001, Welch’s test).

The Regular Registry population has a higher median white coat percentage than the Solid Paint-Bred (Welch’s test, *p* < 0.0001, Table 2 and Figure 1), although the ranges for the two groups are nearly identical. 

Two pigmentation loci, *MC1R* (*E* or *e*) and *ASIP* (*A* or *a*), create the phenotypes of black, bay, or chestnut base coat color. Among these, the chestnut coat color had a significantly higher median percent white coat from that of bay (*p* = 0.0055) and black (*p* < 0.0001, Table 3). 

**Figure 2:**
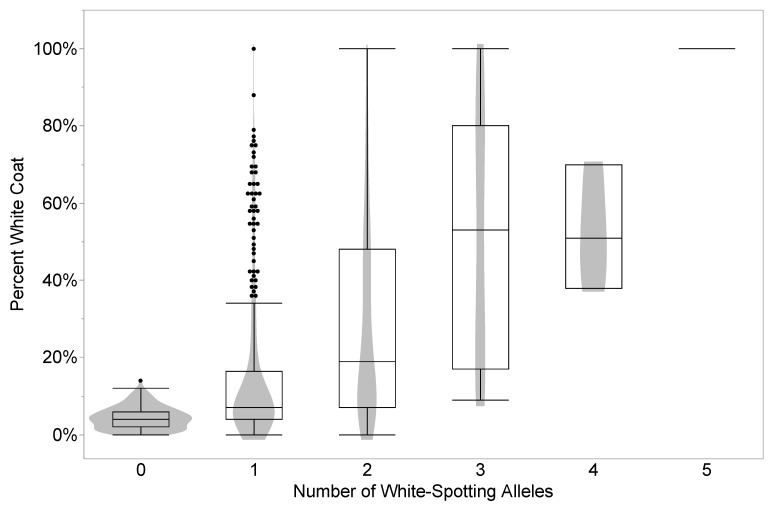
Percent white coat by number of white-spotting alleles. Only one representative possessed 5 white-spotting alleles and had a 100% white coat (*p* = 0.0001, ANOVA).

There is a significant increase in percent white coat as the number of white-spotting alleles increase (% White Coat = 0.0275676 + 0.1344483×# alleles, R^2^ = 0.253, *p* < 0.0001, ANOVA, Table 4 and Figure 2). We observed only one horse with five white-spotting alleles, and it had a 100% white phenotype. 

Among the four predictive models considered here, the simple neural net produced the highest R-square in the validation population of horses, despite ignoring all two-locus epistatic interactions (Table 5). However, even this model could explain at best 55.5% of the variation in the proportion of white coat phenotype. 

## 4. Discussion

Here we documented a quantitative measure of the phenotype resulting from seven individual white spotting alleles in the horse, as well as many multi-locus combinations (Table 1). The “Regular Registry” division in the APHA traditionally captures Paint Horses with “natural paint markings”. In contrast, Solid Paint-Bred horses are intended to have solid coats but with important Paint Horse heritage. As expected, these two groups differ significantly in white spotting phenotype (Table 2 and Figure 1) However, the ranges in phenotype for these two groups overlap almost completely. Horses observed in the Regular Registry but with little white spotting may have entered this group may have white spotting not visible on a side profile view (such as a white ventral belly marking). Additionally, our proportion of white phenotype does not consider the pattern of the distribution of white across the body and would not favor individuals with the smallest qualifying white marking beyond the threshold. The opposite case, the completely white horse possessing five white-spotting alleles, was placed into the Solid Paint-Bred registry as it lacked sufficient contrasting color to qualify for regular registry but would have excellent value as a breeding stock. Exceptional cases like these, and the complex language used to describe the qualitative registration thresholds, can confuse APHA members. 

Alleles controlling base coat pigmentation may exert an effect on white spotting patterns and could therefore be important factors in selecting breeding stock for Paint Horse owners. Indeed, we detected a significant difference in white spotting phenotypes between genotypes for the *MC1R-ASIP* signaling pathway. Thus, although the contrast of white markings on a dark coat may be aesthetically appealing, breeding for the chestnut base coat will increase the phenotypic expression of the white spotting alleles observed here and provide a higher likelihood of exceeding the thresholds defining the APHA Regular Registry and therefore a higher market value. Notably, we did not test the effect of base coat color on the Tobiano phenotype, as the structure of the paracentric inversion responsible for this allele leads to a depression of recombination across a large region on ECA3 and inflates linkage between the *KIT* and *MC1R* loci [2,3]. In this case, any effect on the phenotype from epistasis could not be separated from the effect of linkage between alleles at these two loci. 

We observed evidence of epistasis not just between the base coat color loci and white spotting alleles, but as an additive effect between white spotting loci. Given the small samples for some compound genotypes we instead examined the white spotting phenotype compared to the total number of white spotting alleles, regardless of the number of contributing loci, and could not evaluate interactions between specific loci Nonetheless, a significant trend for an increase in the white spotting phenotype with an increase in the number of non-reference alleles was apparent in this dataset (Table 4 and Figure 2).

Breeders of APHA horses benefit from optimizing the production of foals possessing the desirable and marketable spotting patterns, while minimizing the number of Solid Paint-Bred foals in each crop. With the availability of testing for more than 40 white spotting alleles [9], it is now advantageous for American Paint Horses to be genotyped for white spotting alleles during the registration process, and recent amendments to the APHA rule book now allow horses with minimal phenotypes but desirable genotypes to qualify for the Regular Registry in the APHA. Yet there are no multi-locus prediction tools available for white-spotting phenotypes for the APHA, despite extensive knowledge of the major loci at play, and the simple inheritance patterns demonstrated by these major loci. The four prediction methods tested here could at best provide phenotype predictions with moderate accuracy. This accuracy may not be acceptable in a phenotype prediction tool for practical use by APHA breeders. Consideration of more complex modeling approaches will require larger sample sizes and may not improve prediction accuracy if the remaining variation is due to random events during melanocyte migration across the embryo. Some gains in accuracy may be achieved by genotyping for additional white spotting alleles now known to circulate in the APH population. Additional phenotype “modifying” loci, often hypothesized to exist by APHA members and breeders, that may increase or decrease the proportion of white coat produced by a spotting allele could be discovered by future Genome-wide Association studies using the residual variance in the proportion of white coat phenotype from these predictive models as the phenotype.

The results presented here provide comprehensive and quantitative evaluations of the actions of the observed white spotting loci and can inform policymakers with the APHA as they work to apply genetic selection to help to achieve the goals of the association. Although simple to calculate, one drawback to our white coat percentage phenotype is that it cannot take into account the specific anatomical location or unique patterning within the depigmented regions that are the rationale behind the complex threshold system utilized for registration classification today. Objective and repeatable quantitative evaluation of white spotting patterns from photos might be achieved by application of machine learning approaches to classify pattern quality. These methods could reduce the staff-time devoted to photograph evaluation, saving costs to the association, and may reduce the incidence of human error in the process.

## 5. Conclusions

With over a million horses registered in the APHA, our sample size of 1195 horses, only 975 of which had good quality photos with which to assess phenotype, only represents a small fraction of the entire Paint Horse population and limited observation of many multi-allele genotype combinations at the assayed white spotting loci. However, this study provides quantitative assessment of these phenotypes, and documented interactions among these white-spotting genes and alleles, and their effect on phenotype. Digital means of phenotyping could serve as an objective method to streamline the APH registration process and reduce confusion among owners and breeders seeking to register their horses. A multi-locus prediction model for white spotting phenotypes, as piloted in our results, will require additional sampling to improve accuracy, but could be a valuable tool to the APHA and its members, allowing them to optimize their breeding of quality Paint Horses.

## Figures and Tables

**Table 1:** The white coat percentage phenotype resulting from genotypes and genotype combinations for the 7 white spotting alleles represented by at least two horses in the sample. The reported *p* values tested for difference from the *N*/*N* genotype category bearing none of the nine tested white spotted alleles (* Welch’s test).

	Genotype	Percent White (%)				
		Median	Min	Max	*p* *	N
**Single-locus genotypes**	*N*/*N*	4	0	14	N/A	201
	*W20*/*N*	5	0	20	0.0176	178
	*TO*/*N*	34	4	79	<0.0001	81
	*O*/*N*	8	0	88	0.0001	54
	*TO*/*TO*	36	6	98	<0.0001	46
	*SW1*/*N*	10	5	20	<0.0001	35
	*W20*/*W20*	6	0	15	0.0252	24
	*SB1*/*N*	10	3	27	0.0256	7
	*SW2*/*N*	9	3	22	0.032	7
	*SW3*/*N*	20	16	24	N/A	2
**Multi-locus Genotypes**	*O*/*N*, *W20*/*N*	17	1	92	<0.0001	35
	*TO*/*N*, *W20*/*N*	48	13	82	<0.0001	17
	*SW1*/*N*, *W20*/*N*	9.5	5	20	<0.0001	16
	*O*/*N*, *W20*/*W20*	43	12	96	0.005	7
	*O*/*N*, *SW1*/*N*	13.5	6	19	0.0045	6
	*SW2*/*N*, *W20*/*N*	5	3	14	0.2758	5
	*SW1*/*N*, *SW2*/*N*	17	7	35	0.0323	5
	*TO*/*N*, *O*/*N*	73	65	81	N/A	4
	*SB1*/*N*, *W20*/*N*	43.5	3	99	N/A	4
	*TO*/*N*, *O*/*N*, *W20*/*N*	78.5	53	93	N/A	4
	*TO*/*N*, *SW1*/*N*	50	30	62	N/A	3
	*SW1*/*N*, *W20*/*W20*	10	9	17	N/A	3
	*O*/*N*, *SW1*/*N*, *W20*/*N*	31	14	78	N/A	3
	*O*/*N*, *SB1*/*N*	95.5	93	98	N/A	2
	*O*/*N*, *SW1*/*N*, *W20*/*W20*	60.5	51	70	N/A	2

**Table 2:** Percent white coat phenotypes among the two APHA-determined registration types.

Registry Type	Percent White				
	Median	Std. Dev.	Min	Max	N
Regular	10.00%	0.2377	0%	100%	534
Solid Paint Bred	4.00%	0.0709	0%	100%	226

**Table 3:** Percent white values by the *MC1R* and *ASIP* predicted phenotypes for black, bay, or chestnut (excluding horses carrying a *TO* allele, pair-wise Welch’s Test).

*E* and *A* Predicted Phenotype	Percent White				
	N	Median	Min	Max	Std. Dev.
Black ^a,c^	42	5.00%	0%	96%	0.2256
Bay ^a,b^	141	5.00%	0%	100%	0.1677
Chestnut ^b,c^	417	6.00%	0%	100%	0.14

^a^ *p* = 0.0511, ^b^ *p* = 0.0055, ^c^ *p* < 0.0001.

**Table 4:** Percent white values by the total number of white-spotting alleles.

Total White Spotting Alleles	White Percent				
	Min	Max	Std. Dev.	N	Median
0	0	14	0.0299	198	4
1	0	100	0.1877	365	7
2	0	100	0.2541	170	19
3	9	100	0.3279	23	53
4	38	70	0.1609	3	51
5	100	100	0	1	100

**Table 5:** Fit assessments for each of four phenotype prediction models.

Model	Set	Sample Size	RSquare	RASE ^a^
Neural Net	Training	507	0.7328	0.1091
	Validation	253	0.5551	0.1519
Least Squares	Training	507	0.744	0.1068
	Validation	253	0.4995	0.1610
Bootstrap Forest	Training	507	0.6301	0.1332
	Validation	253	0.4981	0.1640
Generalized Linear	Training	507	0.6403	0.1014 ^b^
	Validation	253	0.4631	0.1300 ^b^

^a^ root average square error, ^b^ root mean square error.

## Data Availability

This work used the same genotype dataset as previously published in Brooks et al. 2020 [2]. Available at Mendeley Data: http://dx.doi.org/10.17632/vxw74dnrtm (accessed on 15 October 2023).
